# Books and Pamphlets Received

**Published:** 1885-04-20

**Authors:** 


### BOOKS AND PAMPHLETS RECEIVED.

*On a New Method of Recording the Motions of the Soft Palate*, by Harrison Allen, M.D., Professor of Physiology in the University of Penusylvania. P. Blakiston, Son & Co., 1012 Walnut St., Philadelphia. 1884.

*The Physician's Daily Pocket Record*, comprising a visiting list, many useful memoranda, tables, etc., by S. W. Butler, M. D. Nineteenth year. New and thoroughly revised, with metric posological table, etc. Edited by D. G. Brinton, M. D. Philadelphia: 1885.

*Home Again!* A Synopsis of a Tour Abroad, by Edward Borck, A. M. M.D., of St. Louis, Mo. Part I, Professional Observations, Hospitals, Internotional Medical Congress, etc., etc. Part II, Sight Seeings, and general useful information.

*Sixteenth Annual Report of the Presbyterian Hospital of the City of New York*, for the year ending September 30th, 1884, with the charter, constitution, by-laws, etc. Organized and Incorporated, 1868.

*The Hygiene of the Nervous System and Mind.* The Relations of the Nervous System to Cholera and its Prophylaxis and Neurotherapy. The Cure and Prevention of Dyspepsia as a Nervous Disease. The Neuropathic Diathesis; its Quarantine and Treatment. By C. H. Hughes, M.D., St. Louis, Lecturer on Psychiatry and Neurology, St. Louis Medical College.

*Address in Medicine*, delivered before the Medical Society of the State of Pennsylvania by W. H. Daly, M.D., of Pittsburg, one of the vice presidents of the American Laryngological Association, Senior Physician for Diseases of the Nose, Throat and Chest to the Pittsburgh Free Dispensary, Pittsburgh, Pennsylvania, etc.

*The Phosphates in Nutrition and the Mineral Theory of Consumption and Allied Wasting Diseases*, an entirely new aud successful treatment, as suggested by the observations and experiments of M. F. Anderson, M. D., Licentiate of the Royal College of Physicians, Edinburgh, Member Royal College Surgeons, England. Presented by Charles H. Phillips, Manufacturing Chemist, New York.

*The Physiological Effects and Therapeutical Uses of Hydrastis*, by Roberts Bartholow, A.M., M.D., LL.D., Professor of Materia Medica and General Therapeutics in the Jefferson Medical College of Philadelphia, etc.

*The Role of Bacteria in Infectious Diseases*, by Henry O. Marcy, A M., M.D., Boston, U. S. A., President of the Boston Gynæcological Society, late President of the American Academy of Medicine, Member of the International Medical Congress, etc.

*A Practical Treatise on Palatable Prescribing*, containing the favorite formulary of the most eminent Medical and Surgical Authorities, collated from their published writings and private records, and embracing a resume of the most eligible prescriptions for the administration of recent additions to the materia medica, by B. W. Palmer, A.M., M.D., author of Favorite Prescriptions of Distinguished Practitioners, Member of the New York County Medical Society, the New York Medico-Legal Society, etc. Detroit: George S. Davis. 1884.

*Does Tobacco Produce Amblyopia?* By W. Franklin Coleman, M.D. M.R.C.S. Eng., Baltimore, Md., Professor of Diseases of the Eye and Ear, Baltimore Polyclinic and Post-Graduate Medical School; formerly Surgeon to the Toronto (Canada) Eye and Ear Infirmary, and Ophthalmic and Aural Surgeon to the St. John (N.B.) Gen. Pub. Hospital.

